# P-271. Evaluation of Admission HIV Screening at a Community Health System

**DOI:** 10.1093/ofid/ofaf695.492

**Published:** 2026-01-11

**Authors:** Bailey Agee, Jeremy J Frens, Emily Sinclair, Cornelius N Van Dam

**Affiliations:** Cone Health, Greensboro, North Carolina; Cone Health, Greensboro, North Carolina; Cone Health, Greensboro, North Carolina; Cone Health, Greensboro, North Carolina

## Abstract

**Background:**

The prevalence of HIV in the US is estimated to be 0.37%, and the Centers for Disease Control (CDC) recommends that all patients between the ages of 13 and 64 undergo HIV testing at least once or annually for patients with ongoing risk factors. To promote adherence with these recommendations, Cone Health implemented several computerized decision support (CDS) modalities including interruptive alerts and dynamic order sets that automatically promote opt-out HIV screening. The purpose of this study is to evaluate the impact of CDS in identifying previously undiagnosed HIV patients at Cone Health.

**Figure 1:**
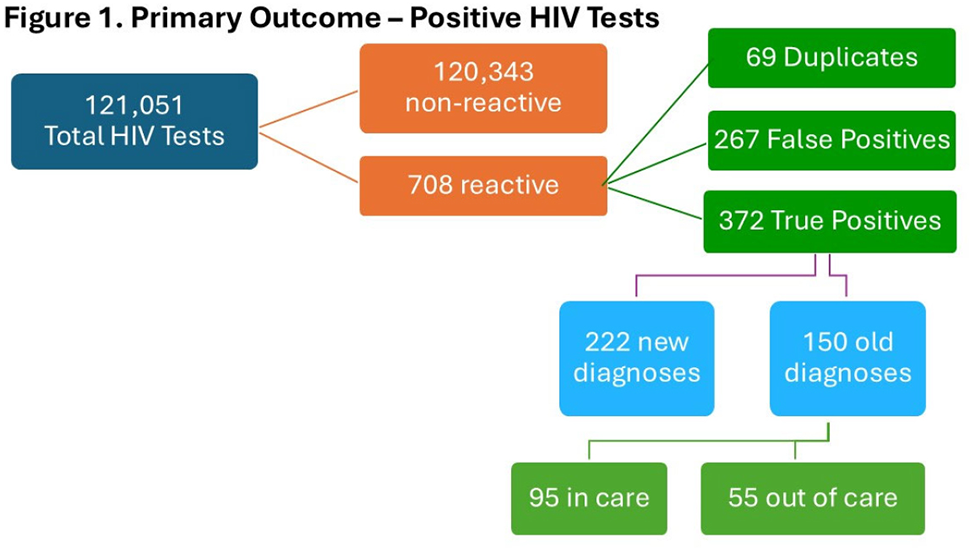
Positive HIV tests

Description of HIV Positive Tests Describing New Diagnoses and Known Positives

Admission Order for HIV Screening

**Figure:**
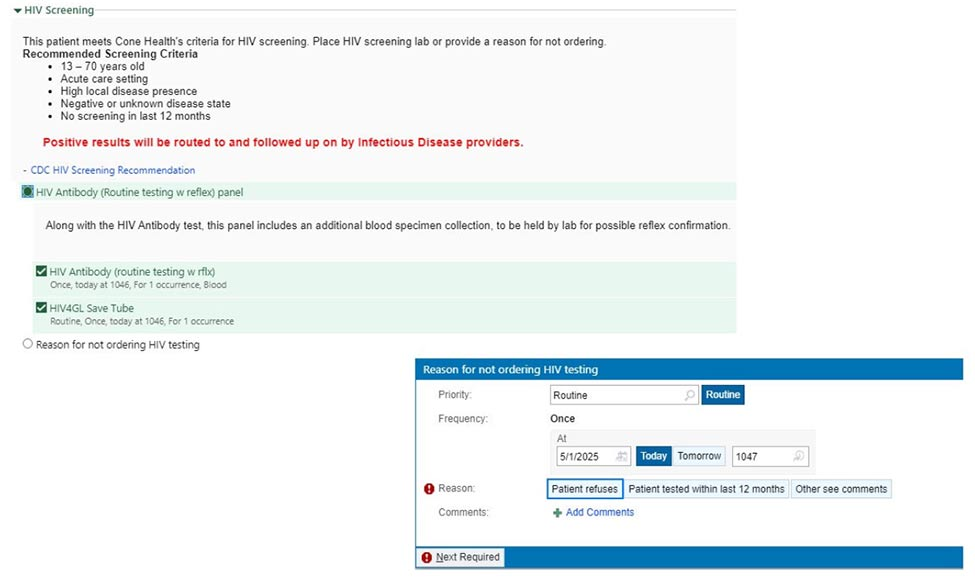

Screenshot of HIV screening orders presented on admission order sets for infection

**Methods:**

A dynamic order set query was added to the general inpatient medical admission order set, automatically selecting HIV screening for patients aged 13 - 70 who have not been screened in the past 12 months and did not have a known HIV diagnosis. Additional prompts to consider HIV screening when testing for sexually transmitted infections (STI) and on hospital admission for an infection diagnosis. Infections Diseases physicians were notified of positive tests through the electronic medical record. Patients with positive tests were offered follow up with a local Infections Diseases clinic for ongoing care. The primary outcome of this study is the number of newly HIV seropositive patients captured via routine HIV screening. Secondary outcomes include the number of false positive tests, indication for HIV testing, number of known positive patients who were tested, connection to care after testing, and HIV treatment initiation.

**Results:**

A total of 121,051 HIV tests were performed from January 1, 2019 through December 31, 2024. Of these, 708 were initially reported as positive, with 372 confirmed cases (0.31%). Of these, 222 were new diagnoses. For patients known to be positive, 55/150 (36%) were not in care. HIV clinic follow-up took place with 244 (65.6%). HIV medication was initiated or resumed in 239 patients (64.2%). The most common reason for testing was STI workup (31.5%) and infection workup (22.3%). Routine screening was able to identify 60 cases (16.1%)

**Conclusion:**

Prevalence of HIV disease was similar to the general US population. CDS to enhance HIV screening was able to identify a significant number of undiagnosed HIV patients and also helped connect previously diagnosed patients to care.

**Disclosures:**

All Authors: No reported disclosures

